# Is “Bed Sharing” Beneficial and Safe during Infancy? A Systematic Review

**DOI:** 10.1155/2014/468538

**Published:** 2014-01-30

**Authors:** Rashmi Ranjan Das, M. Jeeva Sankar, Ramesh Agarwal, Vinod Kumar Paul

**Affiliations:** ^1^Department of Pediatrics, All India Institute of Medical Sciences, Bhubaneswar 751019, India; ^2^Newborn Health and Knowledge Centre (NHKC), Department of Pediatrics, All India Institute of Medical Sciences, New Delhi 110029, India

## Abstract

*Background*. There is conflicting evidence regarding the safety and efficacy of bed sharing during infancy—while it has been shown to facilitate breastfeeding and provide protection against hypothermia, it has been identified as a risk factor for SIDS. *Methods*. A systematic search of major databases was conducted. Eligible studies were observational studies that enrolled infants in the first 4 weeks of life and followed them up for a variable period of time thereafter. *Results*. A total of 21 studies were included. Though the quality of evidence was low, bed sharing was found to be associated with higher breastfeeding rates at 4 weeks of age (75.5% versus 50%, OR 3.09 (95% CI 2.67 to 3.58), *P* = 0.043) and an increased risk of SIDS (23.3% versus 11.2%, OR 2.36 (95% CI 1.97 to 2.83), *P* = 0.025). Majority of the studies were from developed countries, and the effect was almost consistent across the studies. *Conclusion*. There is low quality evidence that bed sharing is associated with higher breast feeding rates at 4 weeks of age and an increased risk of SIDS. We need more studies that look at bed sharing, breast feeding, and hazardous circumstance that put babies at risk.

## 1. Background

In olden days, mothers and babies were separated after birth to prevent infectious diseases and to keep newborns in a safe and controlled environment. The practice of “rooming-in” (keeping the mother and the baby in same room) started when it was realized that the separation had serious implications for the emotional and psychological development of both mothers and newborns [1]. Rooming-in is now recommended by the World Health Organization (WHO) and UNICEF as part of the Baby Friendly Hospital Initiative (BFHI) programme to promote breastfeeding [2].

During rooming-in, the infant is placed close to the mother either by bed sharing, by an attached side-car crib, or by her bedside in a standalone cot. Of these, the “ideal” method would be “bed sharing” in which the newborn is kept on the same bed as that of the mother. The “bed sharing” behavior, however, is controversial in public health parlance. Some consider it a significant risk factor for sudden infant death syndrome (SIDS) and argue for its wholesale elimination [3–5]. Others disagree, finding little or no scientific evidence for an association with SIDS, except among smoking mothers [6–8].

When research into SIDS began in the 1980's, researchers had little idea of why these babies died. In the 1990's, bed sharing related to infant sleeping environment was identified as one of the risk factors. By the end of this decade, a stronger interaction between bed sharing and smoking was observed. One recent study exposed an interaction between bed sharing and alcohol/drugs [9]. This study also partially explained the difference in SIDS rates between cultures where cosleeping is the usual practice. Bed sharing is common in certain cultures where the prevalence of SIDS is high, including the African black populations in the United States, Maori, and Aboriginal populations. Bed sharing is common in certain cultures where the prevalence of SIDS is low, including Asian communities (Japan, Hong Kong, Bangladesh, and those in UK) and Pacific Islander communities in New Zealand. Actually, it is not the bed sharing that distinguishes these cultures, but other factors (e.g., smoking and use of alcohol/drugs) which in conjunction with cosleeping may put infants at risk [9].

With this background, we conducted present systematic review to evaluate the efficacy and safety of bed sharing compared to no bed sharing during infancy, to provide updated evidence to the World Health Organization (WHO) which is in the process of reviewing its recommendations on postnatal care of new born infants and their mothers.

## 2. Methods 

### 2.1. Types of Studies

We intended to include randomized and quasirandomized trials that compared bed sharing of mothers and their infants with no bed sharing. If randomized studies were not available, we planned to include observational studies after applying the following eligibility criteria:

1. cohort studies that enrolled infants in the first 4 weeks of life and followed them up for a variable period of time thereafter were included;
2. case-control studies in which
  - infants being exclusively breastfed at 4–6 wk/3-4 months/6 months of age or those who died of SIDS were the “cases”;
  - the exposure (i.e., bed sharing) occurred in the neonatal period for at least a few “cases”; if this is not explicitly mentioned in the study, we assumed this if the study had reported the exposure status, that is, bed sharing, as a “routine” or “usual” practice;
3. studies that did not include neonates and measured the exposure in the “last sleep” were excluded;
4. cross-sectional studies that measured the association between breastfeeding status at different time points and bed sharing starting from the neonatal period until 1 year of age were included.

We excluded the studies on “rooming-in” if they had not reported bed sharing separately. When there were multiple publications from one study, only the publication with the most relevant information to the systematic review was used.

### 2.2. Exposure or Intervention

Bed sharing of mother and her neonate was the exposure studied. Terminology regarding bed sharing, room sharing, and cosleeping used both in the scientific literature and the popular literature is inconsistent and potentially problematic. For the purposes of this paper, “bed sharing” term was used if the newborn was kept on the same bed as that of the mother.

### 2.3. Outcome Measures and Their Definitions

The primary outcomes were the (a) proportion of infants being breastfed (any or exclusive) at 4–6 wk, 3-4 months, and 6 months of age and (b) proportion of infants dying of SIDS in the first year of life. The secondary outcomes were the incidence of hypothermia, all-cause mortality during neonatal period, and incidence of neonatal sepsis.

Exclusive breastfeeding was defined as an infant receiving only breast milk with no additional foods or liquids, not even water except multivitamin supplements or medications, and any breastfeeding means that an infant receives any amount of breast milk regardless of supplements. SIDS was defined as the unexplained death without warning of an apparently healthy infant usually during sleep. Hypothermia was defined as skin temperature <36.5°C. Neonatal mortality referred to deaths due to all causes occurring in the first 28 days of postnatal life while neonatal sepsis was defined as the clinical syndrome of bacteremia with systemic signs and symptoms of infection in the first 28 days of postnatal life with or without culture positivity.

### 2.4. Search Methodology

The following databases were searched independently by three review authors (Rashmi Ranjan Das, Ramesh Agarwal, M. Jeeva Sankar) using the search terms (newborn OR infant OR neonat*) and (rooming-in OR bedding-in OR bed share OR bed sharing OR cosleeping OR sleeping). The databases searched were and MEDLINE via PubMed (1966–May 2013), Cochrane Central Register of Controlled Trials (CENTRAL, The Cochrane Library, Issue 6, May 2013), and EMBASE (1988–May 2013). Searches were limited to human studies. There were no language restrictions. For further identification of ongoing trials, the website http://www.clinicaltrials.gov/ was searched and relevant trials were screened for eligibility of inclusion in the review. We also checked the cross-references of relevant articles. We treated side-car crib exposure (used in some studies) as no bed sharing.

### 2.5. Data Extraction

Data extraction was done using a standardized data extraction form that was designed and pilot tested *a priori*. Three authors (Rashmi Ranjan Das, M. Jeeva Sankar, and Ramesh Agarwal) independently extracted data from included studies, including year, setting (country, type of population, socioeconomic status, baseline neonatal mortality, baseline practice of rooming-in or bedding-in, gestation, and birth weight of infants), exposure/intervention (bed sharing, routine or last night), and results (outcome measures, effect, significance). Disagreements in extracted data were resolved through discussion.

### 2.6. Assessment of Risk of Bias in Included Studies

Two review authors (M. Jeeva Sankar, Rashmi Ranjan Das) independently assessed the methodological quality of the selected studies. Quality assessment was undertaken using the Newcastle Ottawa Scale (NOS) for observational studies. This scale assesses the quality under three major headings, namely, selection of the studies (representativeness, exposure assessment/control selection), comparability (adjustment for main/additional confounders), and outcome/exposure (adequacy of outcome measured, exposure measured versus self-report) [10]. Any disagreement was resolved through discussion with the third author (Ramesh Agarwal).

### 2.7. Data Analysis

For each outcome measure, the total number of participants and the number of participants experiencing the event and the adjusted odds ratio (aOR) provided by the study authors were extracted. We intended to use only aOR for pooling the results. If adjusted ratios were not provided in a given study, we used the unadjusted OR.

Meta-analysis was done by the generic inverse variance using the user written command “metan” in Stata 11.2 (StataCorp, College Station, TX). We used the natural logarithm converted values of aOR and their confidence intervals (CI) for computing the pooled estimates. The heterogeneity between the studies was quantified by using a measure of the degree of inconsistency in their results (*I* ^2^ statistic). Given that all the included studies were observational and had an inherent risk of heterogeneity between them, we planned to use fixed-effect model for pooling their results irrespective of the degree of heterogeneity. For the outcome of breastfeeding status, we planned to have only one summary result—breastfeeding status at 4 to 6 weeks of age.

## 3. Results

**Figure 1:**
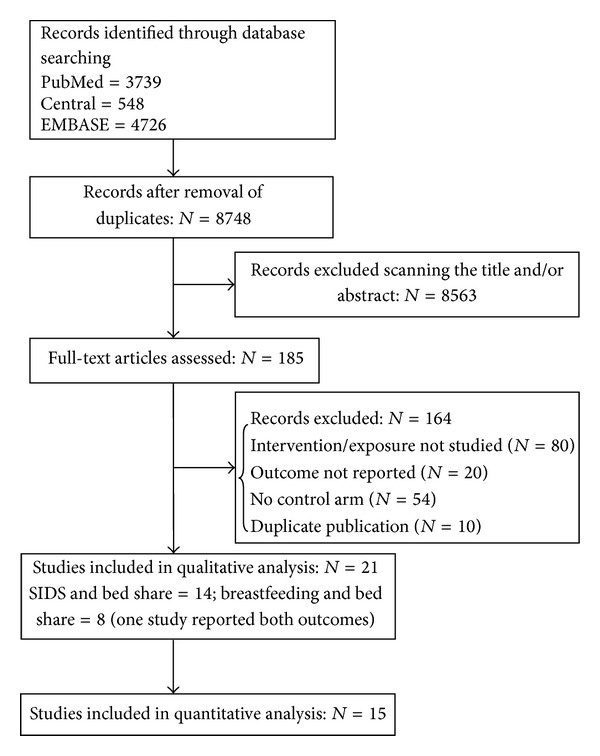
Flow of studies.

We identified 9013 articles, of which 21 were found to be eligible for inclusion (breastfeeding and bed sharing = 8; SIDS and bed sharing = 13; one study reported both the outcomes) (Figure 1) [9, 11–30]. A total of 15 studies provided data for the quantitative analysis (Tables 1 and 2) [9, 13, 14, 19–30]. None of the included studies were randomized trials (RCTs), as we could not find any RCT that specifically studied the intervention (bed sharing). The 13 studies [9, 19–30] that evaluated SIDS and bed sharing were case-control studies while among the 8 studies reporting breastfeeding rate, one was case-control study [12], and the other seven were cross-sectional studies [11, 13–18]. All except three studies [12, 14, 15] were population based. Studies reporting SIDS included 13072 infants and provided data up to 2 years of age while those reporting breastfeeding included 25276 infants and provided data up to 1 year of age. Majority of the included studies were of good quality as per the New Castle Ottawa Scale (Table 3).

### 3.1. Breastfeeding

**Figure 2:**
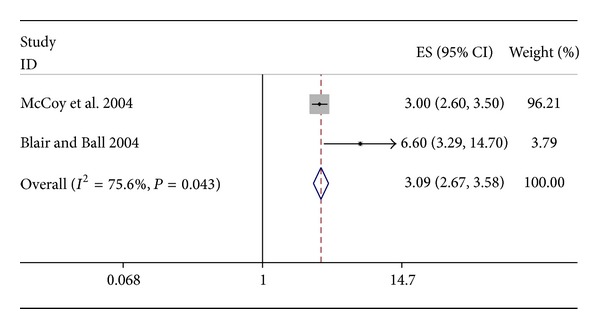
Forest plot: bed share and breastfeeding.

Only one study reported exclusive breastfeeding rates while the others reported *any* breastfeeding rates [15]. Mailed questionnaires were used to collect data in all studies except one which used face-to-face interview [15]. All except the one study were from developed countries [15]. Loss to follow-up rate varied from 2.2% to 50% in the included studies. The risk of bias in these studies was moderate to high (Table 1). Almost all the studies showed a consistent beneficial effect on any/exclusive breastfeeding rates at the three different time points, that is, at 4 to 6 weeks, 3 to 4 months, and at 6 months of age. The effect sizes of the individual studies varied from 1.5 to 6.7 (Table 1). We could pool the results of two studies that reported breastfeeding rates at 4–6 weeks of age [13, 14]. Pooled analysis showed 3-fold odds of being exposed to bed share during the neonatal period in those who were breastfed compared to those who were not (pooled OR 3.09; 95% CI (2.67, 3.58); *I* ^2^ = 75.6%; Figure 2). Since all were observational studies, the quality of evidence as assessed by GRADE criteria was found to be low (Table 4).

### 3.2. SIDS

**Figure 3:**
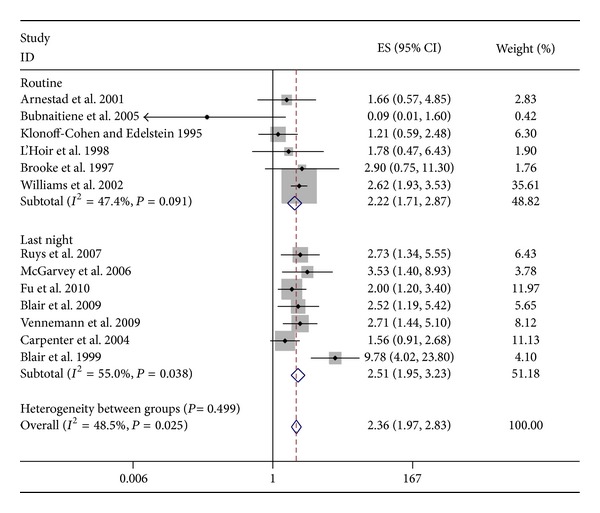
Forest plot: bed share and SIDS.

All except one study that evaluated the association between bed sharing and SIDS were from developed countries [26]. The attrition rate varied from 2.2% to 50% in all but two studies where it was unclear [21, 27]. The attrition rate for both the groups was calculated separately by percentage of those who responded to the total questionnaires distributed for each group. Five studies reported routine or usual bed sharing [9, 12, 20, 26, 28], whereas; rest reported bed sharing on a particular night (last sleep for cases and reference sleep for controls). When we separately studied routine/usual versus last/reference sleep bed sharing, we found similar increase in risk (Figure 3). This means that pattern of bed sharing does not affect the SIDS risk. More than half of the studies showed significant association between bed sharing and SIDS in the enrolled infants (Table 2). Pooled analysis of all 13 studies demonstrated 2.4-fold odds of being exposed to bed share in those who die of SIDS compared to the controls (aOR 2.36; 95% CI (1.97, 2.83); *I* ^2^ = 48.5%; Figure 3). Given that all were observational studies, the quality of evidence as assessed by GRADE criteria was found to be low (Table 4).

### 3.3. Secondary Outcomes

None of the included studies had reported the incidence of hypothermia or sepsis, NMR, among the enrolled infants.

## 4. Discussion

“Bed sharing” has been commonly used interchangeably with terms like “bedding-in” or “cosleeping.” “Bed sharing” is a topic of common interest in regard to its controversial association with sudden infant death syndrome (SIDS) on one hand and breastfeeding on the other hand. There are many reasons that make parents prefer bed sharing with their infants, such as ease in breastfeeding, enjoying the time spent with the infant, comforting the infant in case he gets fussy, and to put him to sleep, attending the infant quickly in case of any mishap or during any illness, promoting love, affection, or bonding, and so forth [12].

Breastfeeding has been proposed to be one of the most prominent reasons for bed sharing. But studies have found difficulties in establishing whether successful breastfeeding leads to bed sharing or bed sharing is because of breastfeeding. Based on the demographic and health surveys conducted between years 2002 and 2008, WHO presented data on indicators assessing infant feeding practices for 46 countries [31]. The data showed that breastfeeding rates are the highest in early infancy, which is also the time with the highest prevalence of bed sharing. In a systematic review based on cross-sectional studies in infancy beyond the neonatal period the authors found a positive correlation between bed sharing and breastfeeding [32]. However, in a longitudinal study a two-way, complex, interdependent, and temporal relationship was found which was itself insufficient to distinguish the independent role of bed sharing in breastfeeding [16]. A common perception is that mothers who breastfeed commonly also bed share frequently in order to avoid any interruption in breastfeeding at night.

The present systematic review demonstrates significantly association between bed sharing during the neonatal period and breastfeeding at 4–6 weeks of age and at 6 month of age. Admittedly, the quality of evidence was low. Although the data generated from observational studies are of low quality, observational studies are still the most common source of information available to SIDS researchers. The studies were also heterogeneous, but we could not carry out either subgroup or metaregression analyses to investigate the cause of heterogeneity because of the small number of studies.

The AAP task force on SIDS recommends that bed sharing should be avoided when the infant is younger than 3 months irrespective of parents smoking status. They also describe that breastfeeding is associated with a reduced risk of SIDS, and if possible, mothers should exclusively breastfeed for 6 months as per the recommendations of the AAP [33]. These statements are perplexing and might be difficult for the parents to follow.

### 4.1. Comparison with the Existing Literature

A recently done meta-analysis on relationship between bed sharing and SIDS included case-control studies with predefined criteria (an adequate definition for SIDS; autopsies performed in >95% of cases; an appropriate description of SIDS ascertainment in the study population; a clear description of the process of control selection; and sufficient data to calculate ORs and 95% CIs or the actual ORs and 95% CIs were provided) [34]. The combined OR for SIDS in all bed share versus nonbed share infants was 2.89 (95% CI 1.99, 4.18). The risk was the highest when maternal smoking was present (OR, 6.27 (95% CI 3.94, 9.99)), and the infant was <12 weeks old (OR, 10.37 (95% CI 4.44, 24.21)). In this meta-analysis bed sharing as an exposure was studied at any time period during infancy and included both routine as well as last night bed share. In contrast, we studied bed sharing as an exposure starting during the neonatal period and continued thereafter for a variable time period. Besides this, we also studied breastfeeding simultaneously which was not done in the abovementioned meta-analysis.

The included studies in present meta-analysis consistently aimed to identify the prevalence of known or potential risk factors for SIDS. Three studies more specifically aimed to investigated bed sharing and SIDS [19, 27, 28]. One study [30] reported bed sharing including those sleeping on the mattress as well as sofa, while another studied bed sharing during the daytime also (this was not used in the present review) [19]. Definitions of sleeping location, whether bed sharing or nonbed sharing, were heterogeneous. For the outcome of SIDS, the most frequently investigated interaction with bed sharing was smoking (most commonly by the mother either during pregnancy or postpartum). We reported the outcomes after adjusting for smoking. An important point that needs mentioned here is that none of the studies reviewed distinguish between planned and accidental bed sharing, a feature that is probably one of the biggest factors involved in the studies identifying an increased risk of this practice.

Only one study from developing country was included in the present meta-analysis [26]. This study reported rate of bed sharing to be 14% in the control subjects, while the SIDS rate in the country was low (0.3 per 1000 live births). Asian countries like Japan and China, though developed, have a lower rate of SIDS despite a higher rate of bed sharing [35, 36]. A bed sharing rate of 37% was reported in Japan in year 2006 when the rate of SIDS was only 0.16 per 1000 live births, and the corresponding figures in China were 24% and 0.16, respectively, during year 2002. Considering these figures, a general recommendation of restricted bed sharing might not be appropriate for developing (including Asian) countries in contrast to developed countries where other known risk factors might play important role.

Published studies from developing countries and particularly from Asian community show a low rate of SIDS in spite of a higher rate of bed sharing. This lower prevalence of SIDS is whether due to the protective effect of breastfeeding or is just a reflection of the demographic that chooses breastfeeding is a matter of debate. One study supported these findings and showed that exclusive breastfeeding at 1 month of age halved the risk, and even partial breastfeeding at 1 month of age reduced the risk [37]. They also found a high incidence of SIDS below 6 months age, and based on these findings they recommended that breastfeeding should be continued until at least 6 months of age when the risk of SIDS diminishes. A recent meta-analysis including 18 studies showed that breastfeeding is protective against SIDS, and this effect is stronger when breastfeeding is exclusive [38]. While both the studies identified a direct protective effect of breastfeeding on SIDS, neither of them examined the impact of bed sharing on the individual outcomes. But studies examining the risk factors for SIDS have found the risk of bed sharing to be so profound that the protective effect of breastfeeding did not significantly influence the magnitude of the risk associated with bed sharing [39–41]. This has been hypothesized to be due to the lifestyle and socioeconomic class that decides both breastfeeding and SIDS rates supported by findings from developed versus developing or Asian countries [40, 42]. Finally, it is actually difficult to isolate breastfeeding and bed sharing from other risk factors related to SIDS.

We did not find any relevant studies, with a contemporaneous comparison, examining the effect of bed sharing in relation to other outcomes of interest (the incidence/prevalence of hypothermia, mortality, and sepsis rate in bed sharing neonates or infants).

### 4.2. Strengths and Weaknesses of Review

The strength of present systematic review is that it included studies having exposure to bed share during neonatal period (and continued thereafter) and addressed both the benefits (breastfeeding) and risks (of SIDS) of bed sharing. But there are some potential limitations that merit attention: (a) methodological issues: all the studies were observational and were conducted mostly in developed country settings; the age group included from the neonatal period and up to 2 years of age, thus making it difficult to generalize the result (as SIDS is meant for during infancy only); the control groups were also variably defined in all the studies (as shown in Table 2); (b) dramatic change in SIDS epidemiology during the last decade [43]; (c) since we included only studies having exposure to bed share during neonatal period and excluded those having exposure during last sleep only, our results cannot be generalized to SIDS as a whole in infancy, as last night bed sharing that happened after the neonatal period has not been captured in our review. Also, we were not able to evaluate the “net” beneficial effect of the intervention as none of the studies had reported both breastfeeding and SIDS rates together.

### 4.3. Further Area of Research

A total of 21 observational studies (breastfeeding and bed sharing = 8; SIDS and bed sharing = 13) were included in the present review. None of the studies reported both the primary outcomes (SIDS and breastfeeding) in bed sharing population, so we were not able to evaluate the “net” effect of bed sharing. Most of the included studies were undertaken more than a decade ago, and the prevalence of bed sharing might have changed as a result of guideline recommendations or societal factors. Bed sharing is a well-known cultural practice in developing countries, but only one study was found to be eligible for inclusion in the review. Control groups were also variably defined in all the included studies. Because prospective studies of SIDS are not possible given the now rarity of these deaths, more detailed retrospective studies that look at bed sharing, breastfeeding, and the hazardous circumstances that put babies at risk are needed.

## 5. Conclusion

There is low quality evidence that bed sharing is associated with higher breastfeeding rates at 4 weeks of age and increased risk of SIDS irrespective of maternal smoking. Due to paucity of studies, it is difficult to predict whether neonates are at a more risk than older infants (>1 month age). We need more detailed studies that look at bed sharing, breastfeeding and hazardous circumstance that put babies at risk.

## Figures and Tables

**Table 1:** Observational studies on bed share and breastfeeding.

S. no.	Study ID/site or country	Design, setting	Study population	Number of subjects	Intervention/exposure	Outcome (effect size)	Comments
1	Flick et al. 2001/USA [11]	Cross-sectional study (within an interventional study), population based	Pregnant women enrolled at 28 wk, contacted at around 8 weeks after delivery	Questionnaire based survey of 218 consecutive infants	Bed share	Breast feeding rate = 61/133 (bed share), 30/85 (no bed share) OR (95% CI): 1.55 (0.86, 2.84)	Any breast feeding (not exclusive breast feeding) at 8 weeks of age. Loss to followup was 2.2%.

2	Ball 2003/United Kingdom [12]	Case-control study, population based	Health infants and mothers, delivered at 36+ weeks. Followed uptill 4 months of age	Cases, 112 Controls, 141	Bed share	Breast feeding rate = 81/112 (case), 54/141 (control) OR (95% CI): 4.2 (2.38, 7.47)	Any breast feeding (not exclusive breast feeding) for ≥1 mo is the definition of the case. Loss to followup was 40%.

3	Blair and Ball 2004/United Kingdom [13]	Data from two studies: one was cross-sectional and the other was longitudinal, population based	Healthy infants and their mothers at home	Data available on 424 subjects	Bed share	Breast feeding rate at 4 weeks = 73/84 (bed share), 113/227 (no bed share) OR (95% CI): 6.66 (3.37, 13.3)	Reported any breast feeding (not exclusive breast feeding rate). Loss to followup was <20%
						Breast feeding rate at 3 months = 58/74 (bed share), 202/350 (no bed share) OR (95% CI): 2.66 (1.43, 5.14)	

4	McCoy et al. 2004/USA [14]	Cross-sectional study, community based (followup at home of institutional births)	Data from the Infant Care Practices Study (ICPS) between years 1995 and 1998. Followup of infants born at selected study hospitals	Data available on 10355 subjects	Bed share	Breast feeding rate at 1 month = 1346/2071 (bed share), 4142/8284 (no bed share) Adjusted OR (95% CI): 3.0 (2.6, 3.5)	Reported any BF rate for >4 weeks. Loss to followup was ~30%
						Breast feeding rate at 3 months = 725/1346 (bed share), 3107/9009 (no bed share) Adjusted OR (95% CI): 3.4 (2.9, 4.0)	
						Breast feeding rate at 6 months = 518/1243 (bed share), 2071/9112 (no bed share) Adjusted OR (95% CI): 3.6 (3, 4.2)	

5	Lahr et al. 2007/USA [15]	Cross-sectional study (random sample survey), population based	Stratified sample of women drawn each month from recently filed birth certificates between years 1998 and 1999	Data available on 1685 subjects	Bed share	Breast feeding rate = 485/584 (bed share), 770/1101 (no bed share) Adjusted OR (95% CI): 2.65 (1.72 to 4.08)	Any breastfeeding for >4 weeks

6	Blair et al. 2010/United Kingdom [16]	Prospective longitudinal study, population based	Infants of all pregnant women residing in the 3 health districts of Avon; age group: birth to 4 years. Bed share was categorized into the following 3 groups: early, late, and constant	Data available on 7447 subjects	Bed share	Breast feeding rate at 6 months = 733/1415 (constant and early bed share), 2111/6032 (no bed share) OR (95% CI): 2.0 (1.77 to 2.25)	Reported only breast feeding rate (not exclusive breast feeding rate). The adjusted OR for bedsharing in the neonatal age group is not known. Loss to followup was 50% loss.
						Breast feeding rate at 12 months = 322/1415 (constant and early bed share), 549/6032 (no bed share) OR (95% CI): 2.94 (2.52 to 3.44)	

7	Tan 2011/Malaysia [17]	Cross-sectional study, facility based	Mother-infant pairs with infants up to 6 months attending health clinics over 4 months in year 2006	Data available on 682 subjects	Bed share	Breast feeding rate = 249/501 (bed share), 45/181 (no bed share) Adjusted OR (95% CI): 1.50 (1.12 to 2.37)	Exclusive breast feeding rate one month prior to interview. Only 20.3% of study infants were <1 mo of age—data for this subgroup is not known. Loss to followup was <5%

8	M$\ddot{\text{o}}$llborg et al. 2011/Sweden [18]	Cross-sectional study, population based	Randomly selected families with infants who had reached 6 months of age	Questionnaire based survey on 8176 families	Bed share	Breast feeding rate = 544/2035 (bed share), 159/2167 (no bed share) Adjusted OR (95% CI): 1.94 (1.56 to 2.41)	Any breast feeding (not exclusive breast feeding) at 6 months of age. Loss to followup was 31.5%

**Table 2:** Observational studies on bed share and SIDS.

S. no.	Study ID/country	Design, setting	Study population	Number of subjects	Intervention/exposure	Outcome	Comments
1	Klonoff-Cohen and Edelstein 1995/USA [19]	Case-control study, population based	Cases: all infants from birth to 1 year of age died of SIDS; controls: matched for birth hospital, sex, race, date of birth, and the same survey	Cases, 200 Controls, 200	Bedshare	SIDS rate = 60/200 (case), 52/200 (control)	No separate data for neonates. The study reported the odds for bed sharing during the daytime also (we did not use that data). OR adjusted for passive smoking. Loss to followup was 25%

2	Brooke et al. 1997/Scotland [20]	Case-control study, population based	Cases: all infant deaths occurring from 7th day of life to 1 year; controls: births immediately before and after the index case in the same maternity unit and matched for the same survey	Cases, 146 Controls, 275	Bed-share	SIDS rate = 11/146 (case), 6/275 (control)	No separate data for neonates available. Other factors studied, GA ≤ 36 wks, BW < 2500 g. Loss to followup was 25%

3	L'Hoir et al. 1998/The Netherlands [21]	Case-control study, population based	Cases: all sudden deaths from 7 days to 2 years of age; controls: matched for date of birth and the same survey	Cases, 73 Controls, 146	Bed-share	SIDS rate = 6/73 (case), 7/146 (control)	Out of 73 SIDS, only 10 happened during the neonatal period. Other factors studied: smoking, alcohol. Data provided for infants who were not exposed to passive smoking

4	Blair et al. 1999/United Kingdom [22]	Case-control study, population based	Cases: all unexpected deaths up to 2 years of age; controls: infants born immediately before and after the index case and matched for the same survey	Cases, 321 Controls, 1299	Bed-share	SIDS rate = 82/321 (case), 189/1299 (control)	Separate data for neonates not available. Other factors studied: smoking, alcohol. Only 23 infants died between 7 and 60 days of life

5	Arnestad et al. 2001/Norway [23]	Case-control study, population based	Cases: all sudden deaths among children between the 2nd week and 3 yrs of age; controls: infants matched for sex and date of birth, randomly picked from the national register and matched for the same survey	Cases, 174 Controls, 375	Bed-share	SIDS rate = 15/174 (case), 24/375 (control)	No separate data for neonates available (out of 174 cases, only 13 died before 2 months of age). Other factors studied: smoking, breast feeding, birth order and weight, mode of sleeping, dummy use, and socioeconomic factors. Adjusted for passive smoking. Loss to followup was 31% (case) and 25% (control)

6	Williams et al. 2002/New Zealand [24]	Case-control study, population based	Cases: all unexpected infant deaths from 29 days to 1 year of age; controls: randomly selected from all births, except home births, and matched for the same survey	Cases, 369 Controls, 1558	Bed-share	SIDS rate = 86/369 (case), 162/1558 (control)	No separate data for neonates available. Other factors studied: smoking, breast feeding. Bed sharing refers to “usual” pattern or last night's sleep is not known. Loss to followup was 10–19%

7	Carpenter et al. 2004/Europe [25]	Case-control study, population based	Cases: all unexplained deaths in the first year of life; controls: randomly selected from the birth records, matched for age and the same survey	Cases, 281 Controls, 1760	Bed-share	SIDS rate = 32/281 (case), 139/1760 (control)	Of the total 700 odd cases, only 57 SIDS occurred in the first month of life. Other factors studied: smoking, alcohol. Information depicted here is for infants whose mothers did not smoke

8	Bubnaitiene et al. 2005/Lithuania [26]	Case-control study, population based	Cases: included <1 year of age group died of SIDS; controls: matched for date of birth, region, and the same survey	Cases, 35 Controls, 145	Bed-share	SIDS rate = 0/35 (case), 20/145 (control)	No separate data for neonates available (only 1 SIDS during the neonatal period). Studied subgroups, GA ≤ 36 wks, BW <2 500 g. Loss to followup was 22.2% in cases

9	McGarvey et al. 2006/Ireland [27]	Case-control study, population based	Cases: all infants from birth to 1 year of age died of SIDS; controls: matched for date of birth, community area, and the same survey	Cases, 259 Controls, 829	Bed-share	SIDS rate = 128/259 (case), 101/829 (control)	Data provided is for infants aged <10 weeks of age. Other factors studied: smoking, alcohol. Loss to followup was 14%

10	Ruys et al. 2007/The Netherlands [28]	Case-control study, population based	Cases: all infants <6 months of age died of cot deaths; controls: infants of the same age groups who participated in a countrywide survey	Cases, 138 Controls, 1628	Bed-share	SIDS rate = 36/138 (case), 151/1628 (control)	No separate data for neonates available. Other factors studied: smoking, breast feeding. Adjusted for breast feeding, age, and passive smoking

11	Blair et al. 2009/England [9]	Case-control study, population based	Cases: all unexpected deaths up to 2 years of age; controls: from the maternity database of one hospital and matched for the same survey	Cases, 79 Controls, 87	Bed-share	SIDS rate = 30/79 (case), 17/87 (control)	Other factors studied: smoking, narcotics, GA ≤37 wks, BW < 2500 g. Neonates accounted for only 15% of SIDS. Loss to followup was 5–14% in both the groups

12	Vennemann et al. 2009/Germany [29]	Case-control study, population based	Cases: all infants from birth to 1 year of age died of SIDS; controls: matched for date of birth and the same survey	Cases, 333 Controls, 998	Bed-share	SIDS rate = 27/333 (case), 28/998 (control)	Data provided is for infants aged <13 weeks of age. Other factors studied: smoking, GA ≤ 37 wks, BW < 1500 g. Adjusted for maternal smoking. Loss to followup was 18–42% in both the groups

13	Fu et al. 2010/USA [30]	Case-control study, population based	Cases: all infants from birth to 1 year of age died of SIDS: controls: matched for birth, race, age, birth weight, and the same survey	Cases, 195 Controls, 194	Bed-share	SIDS rate = 15/195 (case), 6/194 (control)	Reported data for 3 subgroups: <1 mo, 1–3 mo, and >4 mo; only the 1st month data has been used here. Other factors studied: smoking, alcohol. Bed sharing included sleeping on the mattress as well as sofa. Loss to followup was 25%

**Table 3:** Quality assessment of included studies using the NewCastle Ottawa Scale.

Study author, year, country	Selection				Comparability of cases and controls on the basis of the design or analysis	Exposure			Comment
	Is the case definition adequate?	Representativeness of the cases	Selection of controls	Definition of controls		Ascertainment of exposure	The same method of ascertainment of cases and controls	Nonresponse rate	
Carpenter et al. 2004, Europe [25]	Yes, record validation (*)	Yes, obviously representative series of cases (*)	Yes, community controls (*)	Yes, infants matched for age and the same survey area, randomly selected from the birth records (*)	Yes, groups comparable and also adjusted for most potential confounders (**)	Yes, interviews (*)	Yes (*)	Described (*)	Good quality

Blair et al. 1999, United Kingdom [22]	Yes, record validation (*)	Yes, obviously representative series of cases (*)	Yes, community controls (*)	Yes, infants born immediately before and after the index case (*)	Yes, groups comparable and also adjusted for most potential confounders (**)	Yes, interviews (*)	Yes (*)	Described (*)	Good quality

Arnestad et al. 2001, Norway [23]	Yes, record validation (*)	Yes, obviously representative series of cases (*)	Yes, community controls (*)	Yes, infants matched for sex and date of birth, randomly picked from the national register (*)	Yes, groups comparable and also adjusted for most potential confounders (**)	Yes, mailed questionnaire (*)	Yes (*)	Described, 31% and 25% loss for the cases and controls, respectively (*)	Good quality

Bubnaitiene et al. 2005, Lithuania [26]	Yes, record validation (*)	Yes, obviously representative series of cases (*)	Yes, community controls (*)	Yes, infants matched for date of birth and region, randomly picked (*)	Yes, groups comparable. Not adjusted for most potential confounders (*)	Yes, home visits in cases and mailed questionnaire in controls (*)	No	Described only for the cases, 22.2% loss (*)	Good quality

Ruys et al. 2007, The Netherlands [28]	Yes, record validation (*)	Yes, obviously representative series of cases (*)	Yes, community controls (*)	Yes, infants who participated in another survey (*)	No, groups not comparable. Adjusted for most potential confounders (*)	Yes, home visits and direct interview in cases and direct interview in controls (*)	Yes (*)	Not described	Good quality

McGarvey et al. 2006, Ireland [27]	Yes, record validation (*)	Yes, obviously representative series of cases (*)	Yes, community controls (*)	Yes, infants matched for date of birth and population based area, randomly picked from birth register (*)	Yes, groups comparable and also adjusted for most potential confounders (**)	Yes, home interview in both cases and controls (*)	Yes (*)	Described, 14% loss (*)	Good quality

Fu et al. 2010, USA [30]	Yes, record validation (*)	Yes, obviously representative series of cases (*)	Yes, community controls (*)	Yes, infants matched for birth race, age, and birth weight, randomly picked from birth register (*)	Yes, groups comparable. Not adjusted for most potential confounders (*)	Yes, home visits and direct interview in cases and direct interview in controls (*)	Yes (*)	Described, 25% loss (*)	Good quality

Klonoff-Cohen and Edelstein 1995, USA [19]	Yes, record validation (*)	Yes, obviously representative series of cases (*)	Yes, community controls (*)	Yes, infants matched for birth hospital, sex, race, and date of birth, randomly picked from birth register (*)	Yes, groups comparable and also adjusted for most potential confounders (**)	Yes, telephonic interview in cases and controls (*)	Yes (*)	Described, 25% loss (*)	Good quality

Blair et al. 2009/England [9]	Yes, record validation (*)	Yes, obviously representative series of cases (*)	Yes, community controls (*)	Yes, from the maternity database of hospital (*)	No, groups not comparable. Not adjusted for most potential confounders	Yes, home visits and questionnaire in cases and questionnaire in controls (*)	No	Described, 5–14% loss in the two groups (*)	Good quality

Vennemann et al. 2009, Germany [29]	Yes, record validation (*)	Yes, obviously representative series of cases (*)	Yes, community controls (*)	Yes, matched for age, gender, region, and sleep time (*)	Yes, groups comparable and also adjusted for most potential confounders (**)	Yes, home visits and questionnaire in cases and controls (*)	Yes (*)	Described, 18–42% loss in the two groups (*)	Good quality

L'Hoir et al. 1998, The Netherlands [21]	Yes, record validation (*)	Yes, obviously representative series of cases (*)	Yes, community controls (*)	Yes, matched for date of birth (*)	No, groups not comparable. Not adjusted for most potential confounders	Yes, home visits and questionnaire in cases and controls (*)	Yes (*)	Not described	Good quality

Brooke et al. 1997, Scotland [20]	Yes, record validation (*)	Yes, obviously representative series of cases (*)	Yes, community controls (*)	Yes, matched for age, season, and maternity unit (*)	Yes, groups comparable and also adjusted for most potential confounders (**)	Yes, home visits and questionnaire in cases and controls (*)	Yes (*)	Described, ~25% loss (*)	Good quality

Fleming 1996, England	Yes, record validation (*)	Yes, obviously representative series of cases (*)	Yes, community controls (*)	Yes, infants born immediately before and after the index case (*)	Yes, groups comparable and also adjusted for most potential confounders (**)	Yes, home visits and questionnaire in cases and controls (*)	Yes (*)	Described, ~9% loss (*)	Good quality

Williams et al. 2002, New Zealand [24]	Yes, record validation (*)	Yes, obviously representative series of cases (*)	Yes, community controls (*)	Yes, randomly selected from all births, except home births (*)	No, groups not comparable and also adjusted for most potential confounders (*)	Yes, interview based in cases and controls (*)	Yes (*)	Described, 10–19% loss (*)	Good quality

Ball 2003, United Kingdom [12]	Yes, record validation (*)	Yes, obviously representative series of cases (*)	Yes, community controls (*)	Yes, healthy infants and mothers, delivered at 36+ weeks. Followed up till 4 months of age (*)	Yes, groups comparable. Not adjusted for most potential confounders (*)	Yes, sleep logs were used to measure the exposure status (*)	Yes (*)	Described, ~40% loss (*)	Good quality

McCoy et al. 2004, USA [14]	Yes, record validation (*)	Yes, obviously representative series of cases (*)	Yes, community controls (*)	Yes, followup of infants born at selected study hospitals (*)	Yes, groups comparable and also adjusted for most potential confounders (**)	Yes, mailed questionnaire (*)	Yes (*)	Described, ~30% loss (*)	Good quality

Lahr et al. 2007, USA [15]	Yes, record validation (*)	Yes, obviously representative series of cases (*)	Yes, community controls (*)	Yes, stratified sample drawn each month from recently filed birth certificates (*)	Yes, groups comparable. Not adjusted for most potential confounders (*)	Questionnaire based (*)	Yes (*)	Described, 26.5% loss (*)	Good quality

Tan 2011, Malaysia [17]	Yes, record validation (*)	Yes, obviously representative series of cases (*)	Yes, controls attending health facility (*)	Yes, infants up to 6 months attending health clinics (*)	Yes, groups comparable and also adjusted for most potential confounders (**)	Face-to-face interviews using a pretested structured questionnaire (*)	Yes (*)	Described, <5% loss (*)	Good quality

M$\ddot{\text{o}}$llborg et al. 2011, Sweden [18]	Yes, record validation (*)	Yes, obviously representative series of cases (*)	Yes, community controls (*)	Yes, randomly selected families with infants who had reached 6 months of age (*)	Yes, groups comparable and also adjusted for most potential confounders (**)	Yes, mailed questionnaire (*)	Yes (*)	Described, 31.5% loss (*)	Good quality

Flick et al. 2001, USA [11]	Yes, record validation (*)	Yes, obviously representative series of cases (*)	Yes, community controls (*)	Yes, pregnant women enrolled at 28 wk, contacted at around 8 weeks after delivery (*)	Yes, groups comparable. Not adjusted for most potential confounders (*)	Yes, mailed questionnaire (*)	Yes (*)	Described, 2.2% loss (*)	Good quality

Blair and Ball 2004, United Kingdom [13]	Yes, record validation (*)	Yes, obviously representative series of cases (*)	Yes, community controls (*)	Yes, healthy newborn infants and mothers at home (*)	Yes, groups comparable. Not adjusted for most potential confounders (*)	Yes, sleep logs and interviews (*)	Yes (*)	Described, <20% loss (*)	Good quality

Blair et al. 2010, United Kingdom [16]	Yes, record validation (*)	Yes, obviously representative series of cases (*)	Yes, community controls (*)	Yes, infants of all pregnant women residing in the 3 health districts of Avon; age group: birth to 4 years (*)	Yes, groups comparable. Not adjusted for most potential confounders (*)	Yes, mailed questionnaire (*)	Yes (*)	Described, ~50% loss (*)	Good quality

*One point, **two points.

**Table 4:** GRADE evidence table for assessment of bed share versus no bed share for neonates.

Quality assessment							Summary of findings				
Participants (studies) Followup	Risk of bias	Inconsistency	Indirectness	Imprecision	Publication bias	Overall quality of evidence	Study event rates (%)		Relative effect (95% CI)	Anticipated absolute effects	
							With no bed share	With bed share		Risk with no bed share	Risk difference with bed share (95% CI)
Sudden infant death syndrome (critical outcome, assessed with interview based)											
13072 (14 studies) 0–2 years	Serious^1^	No serious inconsistency	Serious	No serious imprecision	Undetected	⊕⊝⊝⊝**VERY LOW** ^1^ due to risk of bias, indirectness	1148/10274 (11.2%)	697/2798 (24.9%)	**OR 2.41** (2.02 to 2.88)	Study population	
										**112 SIDS per 1000**	**121 more SIDS per 1000 **(from 91 more to 154 more)
										Moderate	
											—

Breastfeeding (critical outcome, assessed with interview based)											
10666 (8 studies) 1–12 months	Serious	Serious	Serious	No serious imprecision	Undetected	⊕⊝⊝⊝**VERY LOW** due to risk of bias, inconsistency, indirectness, and large effect	4255/8511 (50%)	1419/2155 (65.8%)	**OR 3.09** (2.67 to 3.58)	Study population	
										**500 BF per 1000**	**256 more BF per 1000 **(from 228 more to 282 more)
										Moderate	
											—

^1^Unclear in most of the studies.
